# Human papillomavirus infection and squamous cell carcinoma of the conjunctiva

**DOI:** 10.1038/sj.bjc.6605466

**Published:** 2009-12-08

**Authors:** C Ateenyi-Agaba, S Franceschi, F Wabwire-Mangen, A Arslan, E Othieno, J Binta-Kahwa, L-J van Doorn, B Kleter, W Quint, E Weiderpass

**Affiliations:** 1 Department of Ophthalmology, Makerere University, PO Box 7072, Kampala, Uganda; 2 Department of Epidemiology and Biostatistics, Karolinska Institute, PO Box 281, Stockholm 171 77, Sweden; 3 International Agency for Research on Cancer, 150 cours Albert Thomas, Lyon 69372, cedex 08 France; 4 Department of Epidemiology and Biostatistics, Makerere University School of Public Health, PO Box 7072, Kampala, Uganda; 5 Department of Pathology, Makerere University, PO Box 7072, Kampala, Uganda; 6 DDL Diagnostic Laboratory, Fonteynenburghlaan 7, Voorburg 2275 CX, the Netherlands; 7 Department of Community Medicine, University of Tromso, Tromso 9037, Norway; 8 Cancer Registry of Norway, PO Box 5313 Majorstuen, Oslo 0304, Norway; 9 Samfundet Folkhälsan, Topeliuksenkatu 20, Helsinki 00250, Finland

**Keywords:** human papillomaviruses, squamous cell carcinoma, conjunctiva

## Abstract

**Background:**

Squamous cell carcinoma of the conjunctiva (SCCC) is associated with HIV-related immunosuppression, but human papillomavirus virus (HPV) is also suspected to have a role. We carried out a case–control study to assess the role of cutaneous and mucosal HPV types in SCCC, conjunctival dysplasia, and their combination (SCCC/dysplasia) in Uganda.

**Methods:**

We compared HPV prevalence in frozen biopsies from 94 SCCC cases (79 of whom were found to be HIV-positive), 39 dysplasia cases (34 HIV-positive), and 285 hospital controls (128 HIV-positive) having other eye conditions that required surgery. Highly sensitive PCR assays that detect 75 HPV types were used. Odds ratios (ORs) and 95% confidence intervals (CIs) were computed, adjusting for, or stratifying by age, sex, and HIV status.

**Results:**

Cutaneous HPV types were detected in 45% of SCCC cases, 41% of dysplasia cases and 11% of controls. Human papillomavirus virus 5 and 8 were the most common types in SCCC, and most often occurred in combination with other types. Associations were observed between SCCC/dysplasia and detection of both single (OR=2.3; 1.2–4.4) and multiple (OR=18.3; 6.2–54.4) cutaneous HPV types, and were chiefly based on findings in HIV-positive patients. Cutaneous HPV infections were rarely observed among HIV-negative patients and the association with SCCC/dysplasia was not significant (OR=2.4; 0.6–9.6) among them. Squamous cell carcinoma of the conjunctiva/dysplasia risk and mucosal HPV types were not associated in either HIV-positive or HIV-negative patients.

**Conclusions:**

We detected cutaneous HPV types in nearly half of SCCC/dysplasia cases and often multiple types (HPV5 and 8 being most common). The role of HIV (confounder or strong enhancer of cutaneous HPV carcinogenicity) is still uncertain.

Since 1980s there has been a substantial increase in the number of reported cases of squamous cell carcinoma of the conjunctiva (SCCC), mainly in sub-Saharan Africa (Poole, 1999; Wabinga *et al*, 2000). The spread of human immunodeficiency virus (HIV) infection has been implicated in the rise of SCCC incidence both in Africa (Ateenyi-Agaba, 1995; Newton *et al*, 2001; Waddell *et al*, 2006) and the United States (Guech-Ongey *et al*, 2008), with an approximately 10-fold increased risk in individuals with HIV/AIDS. Mucosal human papillomavirus (HPV) types were initially suspected to have a role in SCCC onset because of their importance in the aetiology of carcinomas of the anogenital tract and oropharynx (IARC, 2007). However, three small case–control studies from Uganda (Ateenyi-Agaba *et al*, 2004; Tornesello *et al*, 2006; de Koning *et al*, 2008) have suggested that cutaneous HPV types (i.e., types belonging to the genus beta and formerly referred to as *Epidermodysplasia Verruciformis* HPV types; IARC, 2007) are more likely candidates for the causation of SCCC than mucosal HPV types.

This case–control study of SCCC and conjunctival dysplasia was carried out in Uganda to further explore the role of HPV and HIV infection in SCCC risk.

## Materials and methods

### Study participants

From January 2004 to June 2007, 125 and 17 patients admitted to the Departments of Ophthalmology of Mulago Hospital, Kampala, and Jinja Hospitals, Jinja, Uganda, respectively, for eye lesions suspected to be SCCC were invited to participate in this study as cases. None had ever been included in previous scientific studies. Conjunctival biopsies from these patients were divided: one part was frozen and one part was fixed in 10% formalin and embedded in paraffin. Three histological slides were stained with haematoxylin/eosin and initially read by a pathologist (EO) at the Department of Pathology, Makerere University, Kampala, Uganda, and then by two independent pathologists from DDL Diagnostic Laboratory, Voorburg, the Netherlands. In case of disagreement on the presence of SCCC or dysplasia, a fourth expert pathologist in Kampala reviewed the slides. A consensus was achieved on the presence of SCCC in 100 patients. A diagnosis of conjunctival dysplasia was made in the remaining 42 patients.

Eligible controls were patients who had been admitted to the same hospitals as cases for eye conditions other than SCCC or dysplasia, and required surgical intervention. This allowed for the collection of conjunctival biopsies that were subsequently frozen. Controls were frequency-matched by hospital and sex, and had to fall into the same expected age range as cases (i.e., 15–80 years). The most frequent diagnoses were cataract (37%), chalazia (12%), corneal tears (10%), and eye trauma (9%). Patients presenting with pterygium or pingueculum were not included because it was previously suspected that these diseases were associated with HPV infection (Gallagher *et al*, 2001). A total of 309 controls were included. A 10-ml blood sample was collected from cases and controls in a tube containing EDTA. They were also asked to answer a questionnaire providing information on selected socio-demographic and lifestyle characteristics. All participants or their guardians (in the case of minors) signed an informed consent form and the study was approved by the ethics committees of Makerere University and Uganda National Council for Science and Technology.

### HPV testing

Conjunctival biopsy samples from cases and controls were frozen at −80°C and shipped to DDL Diagnostic Laboratory, a certified and dedicated PCR laboratory, which conducted strict separation of clean reagents, DNA isolation, PCR, and post-PCR activities. Five SCCC cases, two dysplasia cases, and 19 controls had no biopsies because of losses, or empty or mislabelled tubes, and were excluded from this study. The DNA was extracted by proteinase K treatment. Each step of the described procedures comprised HPV-negative and -positive controls. For every 10 tissue samples, one DNA isolation-negative control was included. For each run a positive control was included.

Isolated DNA was tested using three different PCR-based assays, one targeting *β*-globin (for DNA quality control), one targeting mucosal HPV types, and one targeting cutaneous HPV types. *β*-globin-negative biopsy samples (one SCCC case, one dysplasia case, and five controls) were also excluded.

Testing for mucosal HPV types was carried out using the short PCR fragment (SPF)_10_-line probe assay (LiPA)_25_-DNA enzyme immunoassay (DEIA) system, (version 1, Labo Bio-Medical Products, Rijswijk, the Netherlands) as described previously (Kleter *et al*, 1998, 1999). Briefly, the broad spectrum SPF_10_ PCR amplifies a 65-bp fragment from the L1 region of the HPV genome. Amplimers were captured onto streptavidin-coated microtitre plates using biotinylated reverse primers. After denaturation of the PCR products by alkaline treatment, a DEIA was used to detect HPV-positive samples. This method is able to detect more than 50 HPV types (van Doorn *et al*, 2006). Amplimers from SPF_10_ PCR DEIA-positive samples were used for subsequent genotyping of 25 mucosal HPV types (high-risk: HPV16, 18, 31, 33, 35, 39, 45, 51, 52, 56, 58, 59, 66, 68, and 70; low-risk: HPV6, 11, 34, 40, 42–44, 53, 54, and 74). The LiPA_25_ SPF_10_ DEIA-positive samples, which were negative on the LiPA_25_ strip, were tested on an additional strip containing probes for 17 additional mucosal HPV types (HPV26, 30, 55, 61, 62, 64, 67, 69, 71, 82, 83, 84, 85, 87, 89, 90, and 91).

Testing for cutaneous HPV types was performed using a PCR reverse hybridisation assay method (The skin [beta] HPV Prototype Research Assay; Diassay BV, Rijswijk, the Netherlands; de Koning *et al*, 2006). It consists of a broad spectrum PCR specifically designed for the amplification of the *β*-HPV genus and targets a fragment of 117 bp from the E1 region of the HPV genome. Combined with the reverse hybridisation assay, the method allows the detection of 25 cutaneous HPV types (i.e., HPV5, 8, 9, 12, 14, 15, 17, 19–25, 36–38, 47, 49, 75, 76, 80, 92, 93, and 96). As no DEIA had been developed for this assay, all amplimers were directly analysed by reverse hybridisation assay.

The sensitivity of PCR assays used in this study for the detection of mucosal HPV types (<100 copies; Kleter *et al*, 1999) was slightly higher than the sensitivity of the PCR assays used to detect cutaneous HPV types (100–1000 copies; de Koning *et al*, 2006).

### HIV testing and AIDS diagnosis

The HIV testing was done at the Nakasero Blood Bank in Kampala, Uganda using an enzyme-linked immunosorbent assay (Murex HIV-1.2.0, Murex Biotech, Dartford, United Kingdom) that is reported to have 99.9% specificity and 100% sensitivity. The HIV-positive findings were confirmed using western blot analysis and all HIV-positive patients received post-test counselling. The diagnosis of AIDS was based on the presence of cytomegalovirus retinitis, cryptococcal meningitis, or skin rash with weight loss.

### Statistical analysis

The association of SCCC, dysplasia or (when findings were similar) their combination (SCCC/dysplasia) with various characteristics, and the positivity for mucosal and cutaneous HPV types was evaluated using unconditional multiple logistic regression equations. Odds ratios (ORs) and corresponding 95% confidence intervals (CIs) were calculated after adjustment for age (as a continuous variable) and sex. All analyses were also adjusted for, or stratified by, HIV status.

## Results

A total of 94 SCCC cases (mean age: 36.7; range: 15–80 years), 39 dysplasia cases (mean age: 32.8; range: 11–50 years), and 285 controls (mean age: 34.0; range: 15–80 years) with HPV results were included in this study. Education, occupation, and cigarette smoking were not related to the risk of SCCC/dysplasia (Table 1). An association (OR=7.3; 95% CI: 4.2–12.4) was seen with HIV infection, which was detected in 85% of cases and 45% of controls. The diagnosis of AIDS was associated with increased risk of SCCC/dysplasia among HIV-positive patients (OR=5.6; 95% CI: 1.5–20.3).

Mucosal HPV types were detected in 6.4, 7.7, and 3.5% of SCCC cases, dysplasia cases, and controls, respectively, and the majority were uncharacterised types (Table 2). Cutaneous HPV types were observed in 44.7, 41.0, and 10.5% of SCCC cases, dysplasia cases, and controls, respectively, and there were few uncharacterised types. Multiple-type infections were detected in 57.1% of SCCC cases, 75.0% of dysplasia cases and 13.3% of controls. The most common types among SCCC cases were HPV5 (*n*=15), 8 (*n*=16), and 24 (*n*=9). Human papillomavirus 5, 14, and 17 were each observed in at least five dysplasia cases. Human papillomavirus 5, 15, and 24 were each observed in at least five controls and only four controls had multiple infections. The few mucosal or cutaneous HPV types detected among HIV-negative cases and controls are shown in parentheses (Table 2).

Table 3 shows the OR values of SCCC, dysplasia, and their combination by positivity for different HPV type(s) after adjustment for age, sex, and HIV status. No association emerged between infection with mucosal HPV types and either SCCC or dysplasia (OR for SCCC/dysplasia=1.0; 95% CI: 0.4–2.7). Among HIV-positive patients the corresponding OR was 1.4 (95% CI: 0.5–3.8 (data not shown)). In contrast, positivity for cutaneous HPV types was associated with significantly increased risks for SCCC/dysplasia (OR =2.3; 95% CI: 1.2–4.4 for single infection and OR =18.3; 95% CI: 6.2–54.4 for multiple infections). Significantly elevated OR values for SCCC/dysplasia were observed for five of the six most common types (i.e., HPV5, 8, 14, 17, and 23), whereas the OR value was also of borderline statistical significance for HPV24. In general, OR values for individual cutaneous types were similar for SCCC and dysplasia except for HPV8 that showed a stronger association with SCCC (OR=39.0; 95% CI: 4.9–310) than with dysplasia (OR=11.3; 95% CI: 1.0–134). The difference between the two OR values was not, however, statistically significant. When cases with well-differentiated and poorly differentiated SCCC were evaluated separately, no differences in the positivity for cutaneous HPV types emerged (data not shown).

The associations of SCCC/dysplasia with cutaneous HPV are shown separately based on the HIV status in Table 4. The OR values very similar to those shown in Table 3 for all study subjects were observed among HIV-positive patients (OR for single and multiple cutaneous HPV infections=2.7; 95% CI: 1.3–5.6 and 15.4; 95% CI: 5.2–45.5, respectively; Table 4). The OR values for individual HPV types in HIV-positive patients (data not shown) were also similar to those in all study subjects. The OR for cutaneous HPV positivity among HIV-negative patients (2.4) had extremely broad CI values (0.6–9.6).

## Discussion

Our study, to the best of our knowledge, is the largest so far on HPV infection and risk of SCCC and conjunctival dysplasia and lends further support to the hypothesis (Ateenyi-Agaba *et al*, 2004; de Koning *et al*, 2008) that cutaneous, but not mucosal, HPV types may be involved in the aetiology of this rare malignancy and its precursor lesion, dysplasia. Our study also showed, however, that cutaneous HPV infection and SCCC/dysplasia were seldom observed in the absence of HIV infection. It remains, therefore, difficult to establish whether the association between SCCC/dysplasia and cutaneous HPV is entirely attributable to the confounding effect of HIV infection, or whether HIV acts as a strong enhancer of the ability of cutaneous HPV types to induce cancer in the conjunctiva. However, we observed a strong difference in the OR values for SCCC/dysplasia between patients who were positive for both HIV and cutaneous HPV (OR=26.0; 95% CI: 12.5–54.1) and those infected with HIV only (OR=4.8; 95% CI: 2.6–8.8) compared with double-negative patients (data not shown). A hint of an association with cutaneous HPV was also observed among HIV-negative individuals, but it was far from being statistically significant. The lack of cutaneous HPV infection in half of SCCC/dysplasia cases detracts from the possibility of the infection to be the necessary cause of SCCC (as mucosal HPVs are for cervical cancer, IARC, 2007), but must be interpreted cautiously because at present even the best tests for cutaneous HPV may have less than perfect sensitivity.

This study does not allow us to draw conclusions on the cancer-causing potential of individual cutaneous HPV types, as a majority of the cases harboured multiple types. In fact, the risk of SCCC/dysplasia seemed to be substantially higher in the presence of multiple than single infections, even after allowance for HIV status. Individually, HPV5 and 8 were the most frequently detected types in SCCC/dysplasia cases, followed by HPV14, 17, 23, and 24. Human papillomavirus 5, 8, 14, and 24 belong to the *β*-1 species and are commonly associated with skin lesions in *Epidermodysplasia verruciformis* and in immunosupressed individuals (IARC, 2007). They are mainly associated with benign lesions, but have also been detected in malignant lesions in both immunosuppressed and immunocompetent individuals. Previous case–control studies on SCCC also identified HPV5 (Ateenyi-Agaba *et al*, 2004; de Koning *et al*, 2008), HPV8 (Ateenyi-Agaba *et al*, 2004; de Koning *et al*, 2008) HPV14 (Ateenyi-Agaba *et al*, 2004; Tornesello *et al*, 2006; de Koning *et al*, 2008), and HPV24 (Ateenyi-Agaba *et al*, 2004) in SCCC biopsies.

Interestingly, we observed a tendency of infection with HPV8 to increase as lesions worsened (from 0.4% among controls to 5.1 and 17.0% among dysplasia and SCCC cases, respectively). This tendency derived entirely from findings among HIV-positive patients. Among HIV-nagative patients HPV8 was dectected in only one SCCC case and no controls, which raises, for the first time, the possibility that a cutaneous type, HPV8, might be a ‘high-risk’ cutaneous type. The classification of HPV types into high- and low-risk types, based on the change in their prevalence from less to more severe neoplastic lesions, has been essential in understanding the role of mucosal HPV infection in cervical cancer (IARC, 2007). A similar classification would greatly help elucidate the role of cutaneous HPV types in SCCC and skin carcinoma. The early genes of HPV8 have been shown to be expressed in the epidermis and to induce spontaneous benign and malignant skin lesions in transgenic mouse models (Schaper *et al*, 2005).

Lack of association of SCCC risk with mucosal HPV types either in HIV-positive or HIV-negative patients in our study is in agreement with most recent studies (for review, see de Koning *et al* (2008)). Notably, of the few infections with mucosal HPV types in our study, most could not be assigned to any of the 42 most common mucosal HPV types for which we performed genotyping. The SPF_10_ PCR we used for mucosal HPV types includes broad-spectrum primers that preferentially amplify mucosal types, but also allows for the amplification of some cutaneous HPV types. It is, therefore, possible that the uncharacterised HPV types detected by assays targeting mucosal types were actually low-viral-copy cutaneous HPV infections (de Koning *et al*, 2008). In fact, all SCCC and dysplasia cases, as well as three out of seven controls with uncharacterised mucosal HPV types also harboured cutaneous HPV types.

The conjunctiva is the only site in equatorial Africans that is not protected from ultraviolet light by heavy pigmentation (Waddell *et al*, 2006). A causal role of heavy ultraviolet light exposure in SCCC onset is strongly supported by the geographical distribution of the disease (IARC, 2007; Guech-Ongey *et al*, 2008), and by the preferential onset of the malignancy in the intra-palpebral zone that is the part of the conjunctiva most heavily exposed to ultraviolet light (Waddell *et al*, 2006). It can probably be assumed that all Ugandans are heavily exposed to ultraviolet light and it is therefore not surprising that no clear association was observed between outdoor occupation and SCCC risk in our study. Education level and cigarette smoking were also unrelated to SCCC risk.

The important strengths of our study include the larger number of cases and controls compared with previous studies (de Koning *et al*, 2008); the exclusion from the control group of any condition suspected to be associated with HPV infection; the availability of frozen biopsy samples for both cases and controls (shown to be better for detection of cutaneous HPV types than paraffin biopsies) (Pfister *et al*, 2003); and the use of highly sensitive and specific PCR assays able to recognise a wide range of mucosal and cutaneous HPV types. In fact, compared with previous studies (Ateenyi-Agaba *et al*, 2004; Tornesello *et al*, 2006; de Koning *et al*, 2008), we observed a higher proportion of SCCC positive for cutaneous HPV types (45%) and fewer uncharacterised cutaneous HPV types. Nevertheless, the family of cutaneous HPV types is broad and heterogeneous (IARC, 2007), so false-negative results cannot be excluded. As expected from what is known about HPV-associated anogenital carcinogenesis (IARC, 2007), dysplasias harboured cutaneous HPV types approximately as often as SCCC.

The weaknesses of this study include lack of information on HPV viral load and HPV subtypes and variants. However, there is at present no evidence that cutaneous HPV subtypes and variants have any clinical relevance. Information on CD4+ count was not available either, but a relationship between SCCC/dysplasia risk and presence of AIDS was observed.

Finally, many more studies exist on cutaneous HPV types and non-melanomatous skin cancer than on SCCC (IARC, 2007). Notwithstanding, the evidence for an association with cutaneous HPV types is stronger and more consistent for SCCC than for non-melanomatous skin cancer (Bouvard *et al*, 2009). Differences in the importance or detectability of cutaneous HPV types between SCCC and non-melanomatous skin cancer may derive from differences in the epithelia of origin (i.e., conjunctiva, the thinnest mucosal membrane in the body, and skin, a stratified squamous epithelium). It is also noteworthy, however, that the control samples used in studies on SCCC (i.e., biopsies from exactly the same location as SCCC) were probably more appropriate than those included in cases–control studies of skin cancer (i.e., a variety of healthy skin samples but mainly plucked eyebrow hairs) (IARC, 2007).

In conclusion, to determine whether any individual cutaneous HPV type is aetiologically important, or simply a correlate of immunosuppression, will require molecular studies of their presence in tumour cells (Quint *et al*, 2009).

## Figures and Tables

**Table 1:** Distribution of 133 cases of SCCC/dysplasia and 285 controls^a^ by selected characteristics and corresponding ORs and 95% CIs

	**SCCC/dysplasia**	**Controls**	**OR (95% CI)^b^**
	***N* (%)**	***N* (%)**	
*Age (years)*			
<35	63 (47.4)	160 (56.1)	
35–44	47 (35.3)	67 (23.5)	
⩾45	23 (17.3)	58 (20.4)	
			
*Sex*			
Male	57 (42.9)	134 (47.0)	
Female	76 (57.1)	151 (53.0)	
			
*Education*			
Illiterate or primary	78 (58.7)	156 (54.7)	1^c^
Secondary or higher	55 (41.4)	129 (45.3)	1.0 (0.6–1.7)
			
*Occupation*			
Indoor	35 (26.3)	66 (23.2)	1^c^
Outdoor	98 (73.7)	218 (76.8)	0.8 (0.5–1.4)
			
*Cigarette smoking*			
No	118 (88.7)	249 (87.7)	1^c^
Yes	15 (11.3)	35 (12.3)	0.9 (0.4–1.9)
			
*HIV status*			
Negative	20 (15.0)	157 (55.1)	1^c^
Positive	113 (85.0)	128 (44.9)	7.3 (4.2–12.4)
			
*AIDS* d			
No	100 (88.5)	125 (97.7)	1^c^
Yes	13 (11.5)	3 (2.3)	5.6 (1.5–20.3)

Abbreviations: CI=confidence interval; OR=odds ratio; SCCC=squamous cell carcinoma of the conjunctiva.

a Some figures do not add up due to missing values.

b Adjusted for age, sex and HIV status, as appropriate.

c Reference category.

d Among HIV-positive only.

**Table 2:** Prevalence of HPV types among cases of SCCC and dysplasia and among controls

	**SCCC (*n*=94)**			**Dysplasia (*n*=39)**			**Controls (*n*=285)**		
**HPV type**	**Single^a^**	**Multiple^a^**	**Total (%)**	**Single^a^**	**Multiple^a^**	**Total (%)**	**Single^a^**	**Multiple^a^**	**Total (%)**
*Mucosal*									
None			88 (93.6)			36 (92.3)			275 (96.5)
Any	6		6 (6.4)	3		3 (7.7)	8 (1)	2	10 (3.5)
16							1	1	2 (0.7)
45	1		1 (1.1)						
52								2	2 (0.7)
53								2	2 (0.7)
83				1		1 (2.5)			
Uncharacterised	5		5 (5.3)	2		2 (5.1)	7 (1)		7 (2.5)
									
*Cutaneous*									
None			52 (55.3)			23 (59.0)			255 (89.5)
Any	18 (1)	24 (1)	42 (44.7)	4	12 (1)	16 (41.0)	26 (11)	4	30 (10.5)
5	2	13	15 (16.0)		8	8 (20.5)	5 (2)	2	7 (2.5)
8	5	11 (1)	16 (17.0)		2	2 (5.1)	1		1 (0.4)
9		1	1 (1.1)		1 (1)	1 (2.6)	1 (1)		1 (0.4)
12		4	4 (4.3)					1	1 (0.4)
14	1	7	8 (8.5)		6	6 (15.4)		3	3 (1.1)
15					1	1 (2.6)	6 (3)	1	7 (2.5)
17	1	3	4 (4.3)	1	4	5 (12.8)			
19		1	1 (1.1)		2	2 (5.1)			
20		4	4 (4.3)		1	1 (2.6)	1		1 (0.4)
21	1 (1)	3	4 (4.3)					1	1 (0.4)
22	1	1	2 (2.1)		2 (1)	2 (5.1)	2 (1)	2	4 (1.4)
23		6	6 (6.4)		2	2 (5.1)		1	1 (0.4)
24	3	6	9 (9.6)	1	1	2 (5.1)	4 (1)	2	6 (2.1)
25	1		1 (1.1)		1	1 (2.6)			
36		1	1 (1.1)						
37		2	2 (2.1)	1		1 (2.6)			
38	1	3	4 (4.3)						
49		2	2 (2.1)						
75		2 (1)	2 (2.1)						
76		1	1 (1.1)	1		1 (2.6)	2 (2)		2 (0.7)
80		1	1 (1.1)		1	1 (2.6)			
92		1	1 (1.1)						
93	1		1 (1.1)						
96		1	1 (1.1)		1 (1)	1 (2.6)			
Uncharacterised	1		1 (1.1)				4 (1)		4 (1.4)

Abbreviations: HPV=human papillomavirus; SCCC=squamous cell carcinoma of the conjunctiva.

a Number of HIV-negative subjects, if any, are shown in parentheses.

**Table 3:**
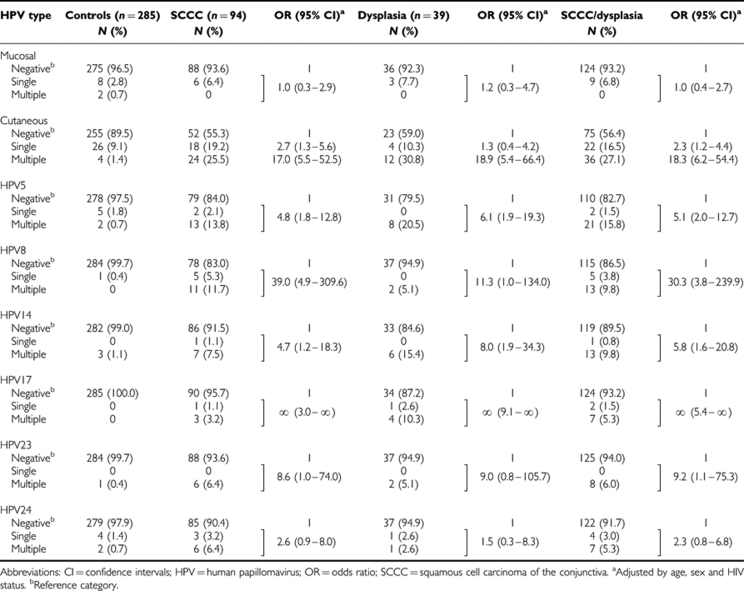
ORs for SCCC and dysplasia and corresponding 95% CIs based on the presence of HPV infection

**Table 4:**
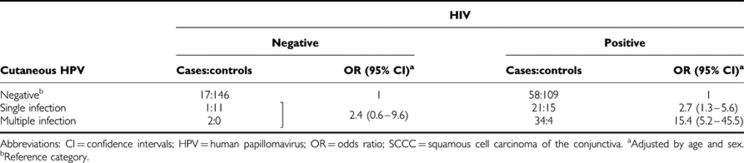
ORs for SCCC/dysplasia and corresponding 95% CIs based on the presence of cutaneous HPV infection and HIV status
